# Supplementary material for: Research on learning achievement classification based on machine learning
Source: PLoS One. 2025 Jun 18;20(6):e0325713. doi: 10.1371/journal.pone.0325713 (PMC12176143; doi:10.1371/journal.pone.0325713)
Supplement: Attachment — Submitted filename: Response to Reviewers.docx [file pone.0325713.s001.docx]

# Responses to Reviewer Comments

Manuscript Number: PONE-D-25-15254

Title: Research on Learning Achievement Classification Based on Machine Learning

Authors: Jianwei Dong, Ruishuang Sun, Zhipeng Yan, Meilun Shi and Xinyu Bi

Dear Editor and Reviewers:

On behalf of all the authors, we express our deep gratitude to you for providing us with the opportunity to revise the manuscript. We acknowledge your valuable and constructive feedback on our work, ‘ Research on Learning Achievement Classification Based on Machine Learning’ (ID: IPM-D-24-00258). We have carefully reviewed the reviewers’ comments and have made every effort to revise the manuscript as suggested. The revised parts are highlighted in red throughout the revised manuscript. Herein we provide a detailed point-to-point response to the reviewers’ comments and concerns.

## Responses to Reviewer #1

We sincerely thank Reviewer #1 for the detailed comments and valuable suggestions about our work. We believe these suggestions must be (and they have been) helpful for us to improve our work. Below we have collated the detailed and valuable comments by reviewer #1, giving our response and the changes we have made to the paper.

**Q1: The study tackles an important and socially impactful problem—predicting student academic performance using machine learning, which has growing significance in the era of data-driven education.**

**Author Response:** We sincerely appreciate the reviewer’s recognition of the significance and societal impact of our research. We agree that predicting students’ academic performance using machine learning techniques is of great importance, especially in the era of data-driven education. Your recognition will inspire us to further improve our work.

**Q2: The proposed Gaussian Distribution-based Data Augmentation (GDO) technique coupled with a Radial Basis Function Network (RBFN) is interesting. However, is GDO a novel method or an adaptation of existing Gaussian-based oversampling techniques like SMOTE variants?**

**Author Response:** We thank the reviewers for their interest in our proposed GDO-RBFN model. The GDO technique we use does draw on the idea of oversampling based on Gaussian distributions, but it is not a simple adaptation of existing Gaussian-based oversampling techniques (e.g., SMOTE variants). Specifically, GDO extends the original dataset by utilising a Gaussian distribution, and in doing so aims to address the problem of limited educational data and category imbalance. Unlike SMOTE and its variants, GDO generates samples based on the statistical characteristics of the data, not only focusing on a small number of class samples, but also emphasising on improving the model’s ability to deal with data with complex features while maintaining the original data distribution properties, especially in terms of non-linear relationships and multi-dimensional features, which makes the approach particularly suitable for the case of educational datasets with a limited number of sample sizes. Therefore, it is the GDO-RBFN that significantly improves the prediction performance of the student performance classification task.

**Q3: The reported accuracy (94.12%) and F1 score (94.46%) are impressive. How does this compare with other baseline models without GDO? Were these improvements statistically significant?**

**Author Response:** We thank the reviewers for recognising the accuracy (94.12%) and F1- score (94.46%) achieved in our study. To assess the effectiveness of GDO, we discussed two experimental settings separately, i.e., with or without GDO amplification. For example, Tables 4-7 show the results of comparisons at multiple baselines without using GDO amplification in different characterisations, and similarly, Tables 8-11 show the results at multiple baselines of comparisons after using GDO amplification. For example, Table 5 compares experimentally with Table 9. The chart numbers are all consistent with the manuscript.

**Table 5. Classify by Personal Issues. The best results are highlighted in red and the second-best results are highlighted in yellow. (%)**

| **Model** | | **Higher Education Students Performance Evaluation Dataset** | | | | **Student Performance** | | | |
| --- | --- | --- | --- | --- | --- | --- | --- | --- | --- |
|  |  | **Accuracy** | **Precision** | **Recall** | **F1-scores** | **Accuracy** | **Precision** | **Recall** | **F1-scores** |
| **ML** | **LR** | 80.00 | 68.34 | 80.00 | 73.64 | 81.52 | 83.25 | 81.52 | 85.29 |
|  | **SVM** | 73.14 | 68.28 | 73.14 | 70.56 | 78.37 | 72.06 | 78.37 | 74.30 |
|  | **RF** | 80.00 | 72.11 | 80.00 | 75.51 | 83.75 | 75.23 | 83.75 | 77.35 |
| **DL** | **MLP** | 77.29 | 68.77 | 77.29 | 72.63 | 79.59 | 70.76 | 79.59 | 75.93 |
|  | **ANN** | 77.29 | 68.70 | 77.29 | 72.51 | 80.12 | 84.08 | 80.12 | 81.54 |
|  | **BILSTM** | 77.24 | 67.93 | 77.24 | 72.19 | 82.94 | 85.66 | 82.94 | 84.78 |
|  | **RBFN** | 78.00 | 68.02 | 78.00 | 72.61 | 92.78 | 92.84 | 92.78 | 92.58 |

**Table 9. Categorize by Personal Questions after Amplification. The best results are highlighted in red and the second-best results are highlighted in yellow. (%)**

| **Model** | | **Higher Education Students Performance Evaluation Dataset** | | | | **Student Performance** | | | |
| --- | --- | --- | --- | --- | --- | --- | --- | --- | --- |
|  |  | **Accuracy** | **Precision** | **Recall** | **F1-scores** | **Accuracy** | **Precision** | **Recall** | **F1-scores** |
| **ML** | **LR** | 77.23 | 77.01 | 77.23 | 76.10 | 77.09 | 76.74 | 72.09 | 78.09 |
|  | **SVM** | 87.50 | 88.76 | 87.50 | 86.93 | 85.69 | 86.93 | 85.69 | 84.71 |
|  | **RF** | 91.11 | 91.76 | 91.11 | 91.12 | 88.16 | 89.34 | 88.16 | 90.42 |
| **DL** | **MLP** | 88.33 | 89.37 | 88.33 | 87.84 | 89.52 | 88.05 | 89.52 | 90.10 |
|  | **ANN** | 87.78 | 88.75 | 87.78 | 87.29 | 85.06 | 86.40 | 85.06 | 86.26 |
|  | **BILSTM** | 87.79 | 88.80 | 87.79 | 82.27 | 86.09 | 89.71 | 86.09 | 89.04 |
|  | **RBFN** | 90.83 | 91.97 | 90.83 | 90.37 | 91.25 | 90.27 | 91.25 | 92.03 |

Regarding statistical significance, the reliability of the synthetic data was verified through variance homogeneity and p-value, and we found that the variance homogeneity and p-value of the characteristics of the overall dataset, personal problem, family issues, and educational habits were higher than 0.05, especially the p-value of the overall dataset, which was 0.8387, suggesting that these improvements were statistically significant and meaningful.

**Q4: It is suggested to add article entitled “Al-Ali et al. Analyzing Socio-Academic Factors and Predictive Modeling of Student Performance Using Machine Learning Techniques” to the literature review.**

**Author Response:** Thank you for your valuable suggestions and we recognise “Analyzing Socio-Academic Factors and Predictive Modeling of Student Performance Using Machine Learning Techniques” article as an important contribution to this research area.

**Author Action:** Based on your suggestion, we have added this section to the literature review and cited it in the body of the manuscript, with the order of the literature remaining the same as in the manuscript. The manuscript is relevantly described as: The more typical K-means clustering and Long Short-Term Memory (LSTM) networks are used to model and predict student performance based on their reported behaviours and preferences [14].

[14] Al-Ali R, Alhumaid K, Khalifa M, Salloum S, Shishakly R, Almaiah M. Analyzing Socio-Academic Factors and Predictive Modeling of Student Performance Using Machine Learning Techniques. Emerging Science Journal. 2024;8:1304–1319. doi:10.28991/ESJ-2024-08-04-05.

**Q5: While the classification accuracy is emphasized, does the study address interpretability of the models? Which features were most influential in predicting academic achievement, and can these be translated into actionable insights for educators?**

**Author Response:** We thank the reviewers for raising the key issue of model explainability. In this study, RBFN was adopted as the core model, student performance is affected by the interaction of multiple factors, compared with the performance of linear models that are difficult to handle, RBFN the model maps input features to a high-dimensional space and is able to capture the complex non-linear relationships between features. Moreover, the RBFN radial basis function and GDO are both based on Gaussian distribution, and the generated new samples can be seamlessly integrated into the local response mechanism of RBFN. As a result, the accuracy of the augmented RBFN is significantly better than other models. In addition, we talk about the classification effects of different feature combinations (overall features, personal problems, family problems, and educational habits) separately by comparatively exploring different feature combination experiments, and we have uncovered the importance of certain features through in-depth exploration of the experimental results. For example, through the data in Table 10, we can find that the most significant improvement in classification accuracy of educational habits features is 93.58% of RBFN accuracy after amplification, which also indicates that these features of educational habits feature group are crucial for achievement prediction. This allows educators to design targeted interventions based on identified characteristics such as study habits, family background, etc., providing educators with prerequisite experience.

**Q6: The paper mentions using variance homogeneity and p-values to evaluate the synthetic data quality. Could more advanced metrics like distribution overlap, Fréchet distance, or t-SNE visualizations be considered for assessing augmentation effectiveness?**

**Author Response:** Thank you for your valuable suggestions, we strongly agree with you and believe that the introduction of more advanced metrics is necessary.

**Author Action:** Following your suggestion, we have also included distribution overlap and Fréchette distance as metrics for assessing the quality of the synthetic data, and the results are summarised in Table 12. Similarly, additional analyses on distribution overlap and Fréchette distance have been conducted in the Discussion section of the manuscript to provide a more comprehensive analysis of the effects of data enhancement. The chart numbers are all consistent with the manuscript. The manuscript is relevantly described as: Since the data were synthesised using GDO, we conducted experiments in order to verify the credibility of the synthesised data, using the Homogeneity of Variance, P-Value, distribution overlap and Fréchette distance as evaluation indicators, and the results of the experiments are shown in Table 12. Starting from the Homogeneity of Variance and P-Value, the Homogeneity of Variance and P-Value of the four categories are higher than 0.05, with the P-Value of the overall dataset being 0.8387, personal problems 0.9210, family problems 0.9075, and educational habits 0.9125. This indicates that there is no significant difference in the homogeneity of variance of the data in different categories, and the hypothesis of homogeneity of variance is valid. significant difference and the assumption of variance chi-square is valid. In terms of distribution overlap and Frechette distance, the distribution overlap values of the four categories are distributed between 0.94 and 0.97, close to 1, indicating that the distributions of raw and synthetic data on each feature are highly overlapped. And the Frechette distance is distributed between 0.02-0.04, all less than 0.05, indicating that the distribution of synthetic data and original data is very similar, suggesting that the synthetic data is of high quality. In summary, the distributions of the synthetic data on these categories were consistent with the original data with high confidence, further verifying the effectiveness of the GDO amplification method in generating credible synthetic data.

**Table 12. Reliability Verification of Synthetic Data.**

|  | **Homogeneity of Variance** | **P-Value** | **Distribution overlap** | **Fréchet distance** |
| --- | --- | --- | --- | --- |
| **Overall dataset** | 0.9177 | 0.8387 | 0.9512 | 0.03 |
| **Personal problem** | 0.9658 | 0.9210 | 0.9705 | 0.02 |
| **Family issues** | 0.9516 | 0.9075 | 0.9400 | 0.04 |
| **Educational habits** | 0.9529 | 0.9125 | 0.9617 | 0.03 |

**Q7: Did the original dataset suffer from class imbalance (e.g., high vs. low-performing students)? How does GDO compare to traditional oversampling techniques like SMOTE, ADASYN, or GAN-based augmentations?**

**Author Response:** In our study, the original dataset suffers from a class imbalance problem, where students’ academic performance, which we measure on a scale of 0-20, is related to course subjects. We study student performance as a separate feature during our experiments, so this imbalance problem does not affect the ability of GDO-RBFN to identify a small number of class samples. GDO has the following advantages over traditional oversampling techniques:

1) GDO generates samples by modelling the statistical characteristics of the data, which effectively reduces noise and improves the quality of synthetic samples.

2)Unlike methods such as SMOTE, GDO can effectively avoid generating overly dense data points, making the generated data more consistent with the actual distribution.

3) GDO can better handle complex multidimensional features, especially in terms of nonlinear relationships and high-dimensional features.

We verified the effectiveness of GDO in generating plausible synthetic data in our experiments by advanced metrics such as variance homogeneity, p-value, distribution overlap, and Frechette distance, as shown in Table 12. The chart numbers are all consistent with the manuscript.

**Q8: The paper acknowledges the limitation in generalization to small datasets or specific student populations. Has cross-validation across multiple cohorts or schools been considered?**

**Author Response:** We acknowledge that there are limitations in the ability to generalise to small datasets or specific student cohorts. In order to further enhance the generalisation ability of the model and to verify its applicability across different cohorts, in fact, a 10-fold cross-validation was conducted during the study, specifically, the original dataset was divided into 10 mutually exclusive subsets, and each time, 9 of them were used as the training set, and the remaining 1 subset as the test set. The process was repeated 10 times to ensure that each sample had the opportunity to participate in the evaluation as a test sample. With this strategy, the problem of limited generalisation ability due to small samples is mitigated to some extent, and the stability and reliability of the model under different data distributions is enhanced.

**Author Action:** We have added a description of the 10-fold cross-validation to the **Results** section of the manuscript. The manuscript is relevantly described as: In order to further enhance the generalisation ability of the model and validate its applicability across different cohorts, a 10-fold cross-validation was carried out during the course of the study, specifically, the original dataset was partitioned into 10 mutually exclusive subsets, and each time, 9 of them were used as the training set, and the remaining 1 subset as the test set. The process was repeated 10 times to ensure that each sample had the opportunity to participate in the evaluation as a test sample. The experiments were based on pytorch to build the RBFN model and Adam was used for learning optimisation.

**Q9: Has there been any consideration of how this model could be deployed in practical educational settings? Could it be integrated into a learning management system (LMS) or used by teachers for early intervention?**

**Author Response:** Various feasible options have been considered on how to deploy the model in real educational settings, but there are certain implementation difficulties, which will be key to the next part of our research. However, we believe that the proposed GDO-RBFN model can be used by teachers for early intervention. By generating personalised reports on students‘ academic performance on a regular basis, teachers can identify students’ learning difficulties and take targeted measures in a timely manner. For example, based on the list of high-risk students predicted by the model, teachers can intervene early to provide additional counselling or resource support.

**Q10: It is also suggested to add articles entitled “Aziz & Elsonbaty. Comparative Study of Different Classification Methods and Winner Takes All Approach” and “Blegur et al. Peer-Assessment Academic Integrity Scale (PAAIS-24)” to the literature review.**

**Author Response:** Again, thank you for your valuable suggestions, both of which you have suggested, ‘Comparative Study of Different Classification Methods and Winner Takes All Approach’ and ‘Peer-Assessment Academic Integrity Scale’, are the most recent articles in this field of study and are important references.

**Author Action:** Following your suggestion, we have added this section to the literature review and cited it in the body of the manuscript, with the order of the literature remaining consistent with the manuscript. The relevant descriptions in the manuscript are:

In addition to this, the concept of academic integrity, i.e. honesty, trust, fairness, respect, responsibility and courage, can be explored in terms of its impact on student achievement [10].

Mahmoud analysed and compared the results of each classifier used, including K-mean, maximum likelihood, support vector machines and neural networks, integrating different types of classification [31].

[10] Blegur J, Subarjah H, Hidayat Y, Ma’amun A, Mahendra A, Mahardika I, et al. Peer-Assessment Academic Integrity Scale (PAAIS-24). Emerging Science Journal. 2024;8:513–526. doi:10.28991/ESJ-2024-08-02-09.

[31]Mahmoud K, Elsonbaty L. Comparative Study of Different Classification Methods and Winner Takes All Approach. Civil Engineering Journal. 2024;10. doi:10.28991/CEJ-2024-010-10-016.

**Q11: Did the authors compare their approach with standard educational prediction benchmarks (e.g., logistic regression, XGBoost, or LSTM)? This would help validate the unique value of the GDO-RBFN approach.**

**Author Response:** Thank you for your valuable suggestion, in fact, in our study, the GDO-RBFN has been compared to several standard benchmark models for educational prediction, including logistic regression (LR) as you mentioned. Although no comparison was made with LSTM, we chose to compare it with the Bidirectional Long Short Term Memory Network (BiLSTM). In addition to this, a variety of prediction baselines such as Support Vector Machines (SVM), Random Forests (RF), Multilayer Perceptrons (MLP), Artificial Neural Networks (ANN), and Radial Basis Function Networks (RBFN) were also compared. In particular, after augmenting the data with the GDO technique, the GDO-RBFN method demonstrated significant advantages, achieving 95.00% accuracy for the overall features and 94.95% for the F1 scores in the students performance evaluation dataset.

**Q12: The conclusion mentions that the RBFN performed best with “educational habit features.” What specifically do these include? Are they related to study frequency, attendance, time-on-task, or something else?**

**Author Response:** Using the students performance evaluation dataset as an example, the educational habits characteristics specifically include: Weekly Study Time, Reading Frequency (Non Scientific Books/Journals), Reading Frequency (Scientific Books/Journals), Attend Seminars/Conferences Related to the Department, etc. Corresponds to rows 17-26 in the dataset characterisation table. They are not related to frequency of study or attendance. Educational habit features are a general term for several categories of features in the dataset, e.g., educational habit features are a general term for the 10 features in rows 17-26 of Table 1. In each dataset, there are several features that represent educational habits, which we call educational habit features.

**Q13: The future direction toward integrating more advanced deep learning and exploring feature engineering techniques is well-aligned with current research trends. Could the authors elaborate on which specific models or techniques they plan to explore (e.g., transformers, attention-based networks)?**

**Author Response:** Dear reviewers, thank you for your interest in the future direction of our research. We fully agree with you that integrating more advanced deep learning models and exploring feature engineering techniques are important current trends in the field of educational data mining and student behaviour prediction. In our future research, we will enhance the ability of RBFN to capture global features by introducing an attention mechanism or hierarchical radial basis functions, and use adaptive parameter optimisation methods to improve the model's adaptability to different data distributions. In addition, the loss function is customised and optimised for the specific needs of educational scenarios. For the selection of data augmentation strategy, we propose to adopt the conditional augmentation strategy to control the generation range and intensity of augmented data, perturb only the non-critical features, and retain the core features to carry out the next step of the study, so as to make my model more suitable for the application of educational classification models.

## Responses to Reviewer #2

We sincerely thank Reviewer #2 for the detailed comments and valuable suggestions about our work. We believe these suggestions must be (and they have been) helpful for us to improve our work. We have collated all the detailed and valuable comments from Reviewer #2 below and have responded point by point.

**Q1: What specific improvements the authors consider regarding the methodology?**

**Author Response:** First of all, I sincerely thank the reviewers for their comments. These comments are very useful for us to improve the paper. We have made the greatest efforts to improve the manuscript based on the suggested content. Next, we will discuss this comment in detail. This paper proposes a new method, GDO-RBFN. Among them, GDO generates new samples based on the statistical characteristics of the data, effectively alleviating the problems of limited data volume and data class imbalance. While RBFN, with its good nonlinear fitting ability and generalization ability, further improves the performance of the model in predicting students’ academic achievements. Compared with traditional oversampling methods such as SMOTE, GDO generates samples based on the statistical characteristics of the data, which can effectively reduce the influence of noise samples, enhance sample diversity, and thereby improve the predictive performance of the model. Furthermore, in this study, GDO-RBFN performed outstandingly in predicting students' academic achievements with its excellent nonlinear fitting ability and generalization ability. It can effectively capture complex nonlinear relationships and maintain good generalization performance with the support of sufficient training data.

**Author Action:** Similarly, we have also made modifications in the original text. For detailed content, please refer to the method section marked in red in the manuscript, or the following: GDO generates new samples based on the statistical characteristics of the data, effectively alleviating the problems of limited data volume and unbalanced data classes. Compared with traditional oversampling methods such as SMOTE, GDO generates samples based on the statistical characteristics of the data, which can effectively reduce the influence of noise samples, enhance sample diversity, and thereby improve the performance of the model. The RBFN, discussed below, further improves the model’s performance in classifying students' academic performance by virtue of its good nonlinear fitting and generalisation abilities.

**Q2: Kindly enrich the literature review of this paper by citing additional references related to the topic addressed: Quantized Iterative Learning Control of Communication Constrained System with the Encoding and Decoding Mechanism, Transactions of the Institute of Measurement and Control; ADP-Based Prescribed-Time Control for Nonlinear Time-Varying Delay Systems With Uncertain Parameters, IEEE Transactions on Automation Science and Engineering; Saturated-threshold event-triggered adaptive global prescribed performance control for nonlinear Markov jumping systems and application to a chemical reactor model, Expert Systems with Applications; It could be the object of a brief consideration focused on the advances on the topic and make relation with this paper, which have to be discussed to indicate the contribution in the Introduction section, and in that way point out other contemporary approaches and possibilities.**

**Author Response:** We thank the reviewers for their comments on our work. We have included the following references in the manuscript that are relevant to the topic under discussion and explore the relationship to the direction of the study than the present one, thus eliciting the feasibility and research implications of our approach, as detailed below.

**Author Action:** Here is the relevant description from our manuscript, with the order of the literature remaining consistent with the manuscript.

ADPPTC can accurately complete tasks within a specific time frame, ensuring optimality and specified time stability.[35]

Since the real situation contains several unknown factors, failure to select appropriate robustness measures may lead to performance degradation or instability of the system [39].

[35] Zhang Z, Zhang K, Xie X, Stojanovic V. ADP-Based Prescribed-Time Control for Nonlinear Time-Varying Delay Systems With Uncertain Parameters. IEEE Transactions on Automation Science and Engineering. 2025;22:3086–3096. doi:10.1109/TASE.2024.3389020.

[39] Song X, Sun P, Song S, Stojanovic V. Saturated-threshold event-triggered adaptive global prescribed performance control for nonlinear Markov jumping systems and application to a chemical reactor model. Expert Systems with Applications. 2024;249:123490. doi:https://doi.org/10.1016/j.eswa.2024.123490.

**Q3: Authors should argue their choice of the performance evaluation indicators.**

**Author Response:** The core reason for the accuracy rate lies in its conciseness and the ability to summarize the overall performance, which is particularly suitable for educators to quickly evaluate the general performance of the model. However, in practical applications, it is necessary to determine whether additional indicators need to be supplemented by considering whether the data distribution is balanced and the cost of error types such as missing high-risk students. Therefore, we simultaneously introduce the precision rate, recall rate and F1 score for analysis. The reason why the accuracy rate is used as the core evaluation index lies in that it directly reflects the model’s ability to avoid misjudging and overestimating students’ grades, that is, to reduce “false positivity”. Ensuring that the model’s prediction results are reliable enough in the positive direction is crucial for the rational allocation of educational resources and the effectiveness of intervention strategies. In educational classification models, choosing Recall as the core evaluation metric can ensure that the model can identify all high-performing or high-risk students who truly need attention to the greatest extent, thereby avoiding omissions and reducing “false negatives”. High accuracy may be accompanied by a low recall rate, which may lead to the omission of some truly high-performing students. In order to consider the comprehensiveness of the model evaluation indicators, this study comprehensively adopts the F1 score. The F1 score integrates accuracy and recall rate, balancing the model’s performance in reducing false positives and missed positives, and is particularly suitable for environments with limited educational resources. We also uses weighted averages to weight the indicators based on the proportion of each category in the dataset to ensure the fairness and comprehensiveness of the evaluation.

**Q4: Have the authors experimented with other sets of values? What are the sensitivities of these parameters on the results?**

**Author Response:** We would like to thank the reviewers for their detailed review of our work and helpful suggestions for the improvement of our paper. In this study, we have analysed and explored the value of k (number of nearest neighbours) in GDO in detail, and finally modified k through several experiments, and discussed the reasons for the different choices with different results. We also did comparison experiments with different numbers. Please refer to the tables and the following section for more details. Also in section **Sensitivity Parameter Analysis** of the article we have carried out an analysis of the sensitivity of different feature combinations to the oversampling rate.

**Author Action:** The following additions were made to the **Sensitivity Parameter Analysis** section of our manuscript to explore the different effects of the choice of k in the GDO method on the results in the grouping of educational habitus characteristics.

k represents the number of nearest neighbors considered when calculating the weights of minority class samples. The selection of the k value has a significant impact on the weight calculation of minority class samples. A smaller k value may cause the generated samples to be overly concentrated, while a larger k value may introduce noise. During this research process, we adjusted parameters such as k through experimental comparison to optimize the performance of the GDO method, thereby achieving the generation of higher-quality minority class samples and solving the problem of class imbalance. The following are the results of the comparative experiment:

**Table 13. Effect of k-value in GDO on the accuracy of classification of educational habitus features.**

| **Model** | **k** | **Higher Education Students Performance Evaluation** | | | | **Student Performance** | | | |
| --- | --- | --- | --- | --- | --- | --- | --- | --- | --- |
|  |  | **Accuracy** | **Precision** | **Recall** | **F1-scores** | **Accuracy** | **Precision** | **Recall** | **F1-scores** |
| **RBFN** | k=4 | 92.16 | 93.65 | 93.14 | 92.37 | 94.24 | 93.22 | 93.24 | 92.11 |
|  | k=5 | 94.12 | 94.60 | 94.12 | 94.46 | 95.20 | 95.87 | 95.20 | 95.67 |
|  | k=6 | 93.14 | 94.22 | 93.06 | 91.75 | 94.88 | 95.06 | 94.85 | 91.39 |

The experimental comparison in Table 13 reveals that when the value of k is 5, all the evaluation indexes of educational habit feature classification are optimal for both datasets. We can optimise the performance of the GDO method by adjusting the size of k.

**Q5: Explain the feasibility of the results from the implementation and computational point of view.**

**Author Response:** We thank the reviewer for the comments on our work. We considered the number of parameters and the computational complexity to discuss the feasibility of the framework. The number of parameters in GDO is relatively small and is mainly determined by the number of neighbors k (5), the covariance coefficient $\alpha$(0.2), and the target oversampling rate p (1). These parameters have limited influence on the computational load. Its computational load mainly focuses on nearest neighbor calculation and new sample generation. The implementation is relatively simple, relying on nearest neighbor search and random number generation, and it is also available on large-scale datasets.

The parameters of RBFN are determined by the connection from the input layer to the hidden layer (M×(D+1)) and the connection from the hidden layer to the output layer (M×C+C), where M (300) is the number of neurons in the hidden layer, D (32) is the input dimension, and C (128) is the output dimension. Its computational load mainly comes from forward propagation and the training process. Therefore, GDO-RBFN is simple to implement in terms of computing resources and optimization strategies, suitable for quickly handling class imbalance problems, capable of effectively handling nonlinear relationships, and suitable for modeling data patterns.

**Q6: Authors should clearly define what quantities must be specified to begin the algorithm. Did the authors consider how many initial conditions affect the outcome?**

**Author Response:** We sincerely thank the reviewers for pointing out this fact. We have added a detailed explanation of the experimental setup and the reasons for setting it up this way. For specific content, please refer to the red-marked content in **Experiment** section of the manuscript and the following text.

**Author Action:** In this experiment, in order to improve the running rate and performance of the model, when we set the learning rate lr of RBFN, the default values of the two datasets were 0.005, the embedding dimension was set to 32, and the number of neurons in the hidden layer was 300. For the GDO module, we adopt a neighbor number of 5, a covariance coefficient of 0.2, and a target oversampling rate that are dynamically adjusted according to different feature groups.

**Q7: The paper does not explicitly discuss the limitations of the proposed algorithm. Every algorithm has its shortcomings, and it would be helpful to include a section discussing potential drawbacks or scenarios where considered approach might not perform optimally.**

**Author Response:** We are very grateful to the reviewers for their comments on us. Based on the reviewers’ comments, we added **Limitations** section to analyze our failure cases and the limitations of the experiment. Finally, we summarized the prospects and the main directions of the subsequent research. The detailed content can be found in this Section of the manuscript or the following text.

**Author Action:** Through the exploration in Tables 4-8, we found that the research results of grouping and classifying according to different features revealed that the RBFN method we proposed did not lead in all datasets and evaluation metrics. This might be due to its weak local sensitivity and adaptability to high-dimensional data, resulting in not leading comprehensively in all scenarios. The data characteristics also have the factor of insufficient matching degree with the local kernel function of RBFN. The fact that we adopted hyperparameters without optimizing for different datasets might also be one of the reasons why RBFN has not achieved comprehensive leadership.

Secondly, combining Table 6 and Table 10, the ranking of our GDO-RBFN decreased instead after being augmented by GDO. Although GDO generates new samples through Gaussian distribution, the generated samples may still have certain similarities, resulting in insufficient sample diversity. This may affect the model’s generalization ability for minority class samples. Furthermore, the synthetic samples generated by GDO may deviate from the true distribution characteristics of the original data, causing the model to learn false patterns on the enhanced dataset, which instead reduces the generalization ability on the original test set. This is particularly sensitive in educational data, where student characteristics often have complex underlying structures.

In the future, we will enhance the ability of RBFN to capture global features by introducing an attention mechanism or hierarchical radial basis functions, and use adaptive parameter optimisation methods to improve the model’s adaptability to different data distributions. In addition, the loss function is customised and optimised for the specific needs of educational scenarios. For the selection of data augmentation strategy, we propose to adopt the conditional augmentation strategy to control the generation range and intensity of augmented data, perturb only the non-critical features, and retain the core features to carry out the next step of the study, so as to make my model more suitable for the application of educational classification models.

**Q8: Each reference has to be double verified, and also reference writing style needs to be uniform. It is essential to review all references, fill in any gaps with Volumes, Issues, and Pages, and revise any inaccurate information. Further, for current references that aren't yet listed in Volume and Issue, DOI numbers must also be added.**

**Author Response:** In response to the questions raised by the reviewers regarding the format of our references, we carefully re-examined the references in the manuscript and made more rigorous revisions. The format of the revised references is also more standardized.
